# Freezing tolerance and recovery of arbuscular-mycorrhizal and non-mycorrhizal *Thuja occidentalis*

**DOI:** 10.1093/treephys/tpag048

**Published:** 2026-04-23

**Authors:** Virpi Virjamo, Tapani Repo, Tarja Lehto

**Affiliations:** School of Forest Sciences, University of Eastern Finland (UEF), P.O. Box 111, FI-80101 Joensuu, Finland; Natural Resources Institute Finland, Yliopistokatu 6, FI-80100 Joensuu, Finland; School of Forest Sciences, University of Eastern Finland (UEF), P.O. Box 111, FI-80101 Joensuu, Finland; Natural Resources Institute Finland, Yliopistokatu 6, FI-80100 Joensuu, Finland

**Keywords:** arbuscular mycorrhiza, cold, conifer, frost, phosphorus, recuperation

## Abstract

Mycorrhizal symbiosis increases nutrient uptake of the host plant, and it can also improve their stress tolerance. Roots are considered the most frost-sensitive plant parts. However, the freezing tolerance of mycorrhizas, and especially arbuscular mycorrhizas (AM), is poorly understood. Here, we studied the freezing tolerance and recovery of AM and non-mycorrhizal (NM) *Thuja occidentalis* (L.). After a simulated summer and autumn, whole-plant freezing tests were done using seven exposure temperatures from 5 °C to −45 °C. Then freezing damage of needles was assessed by relative electrolyte leakage. The seedlings were kept for 2 weeks in long-day recovery conditions with day temperature either 10 °C or 22 °C, and then visual damage, shoot and root mass, nutrient concentrations and mycorrhizal colonization were analyzed. Before the frost exposure, AM plants had higher phosphorus (P) concentrations and similar growth to NM plants. Needle freezing tolerance was −23 °C (corresponding to lethal temperature for 50% of specimens) and was not affected by AM. Visual investigation after the recovery period showed a similar result. Lower foliar nitrogen (N) concentration and root mass in seedlings exposed to −18 °C in both AM and NM plants suggests that fine-root damage had taken place already before −18 °C. Recovery in 22 °C increased nutrient uptake and growth only in seedlings exposed to +5 °C and −5 °C, but specific root length increased also after −18 °C. The AM plant shoots grew less than NM ones during the recovery period. Arbuscular mycorrhizas increased foliar N during recovery in all non-lethally exposed seedlings, and P concentrations in seedlings exposed to +5 °C and −5 °C. This was due to a concentration effect by the lower dry mass increment. These results suggest that the functioning of AM fungi can be limited by short growing seasons or in cold soil conditions, which may affect their distribution in cold regions.

## Introduction

Mycorrhizal symbiosis between plants and fungi is crucial for nutrient uptake in most terrestrial vascular plants. It has been proposed that mycorrhizas could increase plant tolerance against different abiotic and biotic environmental stresses such as drought, frost and pathogens (Augé 2001, Borowicz 2001), but their role in adaptation to different temperatures is still poorly understood (Tibbett and Cairney 2007, Korhonen et al. 2019).

The major types of fungi forming mycorrhizal symbiosis are arbuscular mycorrhizal fungi (AMF) and ectomycorrhizal fungi (EMF), which differ in their root colonization patterns, ability to utilize different forms of soil nitrogen (N) and phosphorus (P), their environmental requirements, and geographical distribution (Read and Pérez-Moreno 2003, Phillips et al. 2013). Globally, plant species forming arbuscular mycorrhizas (AM) are the most abundant, but most of the major tree species in the boreal and cool-temperate zone form ectomycorrhizas (EM; Smith and Read 1997). This has been linked to low soil pH and N-limited growth conditions in boreal zone, while P limitation is more common in drier and warmer regions dominated by AM (Read 1991, Read and Pérez-Moreno 2003). However, we have suggested that the dominant role of EM symbiosis in the northern areas is also related to better functioning in cool growing-season soil conditions and survival of harsh winters (Lehto and Zwiazek 2011, Kilpeläinen et al. 2016, 2020).

In the coming years, atmospheric CO_2_ will continue to increase and the temperatures will increase especially in the northern latitudes (IPCC 2014). During the growing season both CO_2_ fertilization and warming generally benefit formation of AM symbiosis (Heinemeyer and Fitter 2004, Büscher et al. 2012, Dong et al. 2018). Paradoxically, warming air temperature may mean colder soil conditions during wintertime in northern forests (Groffman et al. 2001). Snow is a remarkably good thermal insulator, and with less snow, there will be repeated soil freezing and thawing cycles (Venäläinen et al. 2001). Additionally, extreme weather events are expected to be increasingly common. Frost is especially harmful for fine roots because they are the most vulnerable part of plants to freezing damage (Hawkins et al. 2021), and they are located mostly in the topsoil which is subject to repeated freezing and thawing.

There are few studies on frost survival of mycorrhizal fungi and the effects of mycorrhizas on root freezing tolerance (Korhonen et al. 2019). This is true especially for AMF which are less common in the boreal forest zone, and accordingly studied typically in milder conditions than EMF (Tibbett and Cairney 2007). Soil-borne propagules of both EMF and AMF can survive even extreme temperatures down to −130 °C, but delayed AM formation and reduced P accumulation after soil exposure indicated that mainly spores survived the lowest temperatures rather than vegetative mycelium (Kilpeläinen et al. 2016). By contrast, pure cultures of some ectomycorrhizal species grew after treatment at −48 °C (Lehto et al. 2008). Ectomycorrhizal fungi are known to increase the concentrations of soluble carbohydrates in their mycelia at low temperatures, possibly acting as cryoprotectants in cellular freezing (Tibbett et al. 2002). Yet, EM did not increase the freezing tolerance of Scots pine (*Pinus sylvestris* L.) roots or whole seedlings in the short-term compared with non-mycorrhizal (NM) ones (Korhonen et al. 2015, 2019). Although AM mycelium was shown to survive winter in the soil and remain infective, the proportion of metabolically active hyphae reduced when soil was frozen (Addy et al. 1994, 1997). In vitro, some of the metabolically active AM hyphae have been shown to survive treatment at −12 °C (Addy et al. 1998), but there are no studies of the freezing tolerance and survival of AM fungi in symbiosis at lower temperatures.

Our aim was to assess the frost tolerance as well as subsequent recovery of NM and AM *Thuja occidentalis* L. seedlings, which were raised for one growing season followed by a short-day and low-temperature acclimation period. Intact potted seedlings were exposed to freezing tests. We used seven different temperatures between 5 and −45 °C, and after the freezing tests two different recovery temperatures (10 °C and 22 °C) in long-day condition, mimicking cold and warm spring conditions after winter. Using both mycorrhizal and NM *T. occidentalis* we aimed to resolve if arbuscular mycorrhizal symbiosis affect *T. occidentalis* frost tolerance, and the capacity of the seedlings to recover growth and nutrient uptake after frost exposures. We hypothesize that: (i) nutrient uptake, especially P uptake, and growth is higher in mycorrhizal seedlings compared with NM seedlings after frost exposure, (ii) growth, nutrient uptake and mycorrhizal colonization suffer from severity of frost treatment, and (iii) after frost exposure, growth, nutrient status and mycorrhizal colonization are reduced more at low than warm recovery temperature.

## Materials and methods

### Growth medium, inocula and plant material

Eastern white cedar (*Thuja occidentalis* L.), as other Cupressaceae species, forms obligately AM symbiosis (Brundrett et al. 1989, Bainard et al. 2011). The natural distribution range of the species falls between 40°N and 50°N in the eastern half of the North America (Hofmeyer et al. 2009), and it is commonly grown as an ornamental in northern Europe.

The growth medium was 4:6 (v/v) mixture of commercial peat (Luonnonturve, unfertilized natural peat pH 4.2, Kekkilä, Vantaa, Finland) and perlite (Agra-perlite RHP, Pull Rhenen, TX Rhenen, Netherlands). Peat was ground through a 6-mm sieve and 1.3 g dm^−3^ of CaCO_3_ was mixed to peat to set the pH between 5 and 6. The mix was sterilized by autoclaving.

The AM and NM treatments were achieved by using living or sterilized inoculum in the growth media. Although autoclaving does not eliminate all viable spores, it reduces their abundance substantially, making the risk of contamination low (e.g., Endlweber and Scheu 2006). Possible contamination from sterilized growth medium or inoculum was controlled with mycorrhizal analyses from random pots in the NM treatment. Soil from 0 to 30 cm depth next to known AM- forming tree species (e.g., *Sorbus aucuparia* L., *Acer platanoides* L. and *T. occidentalis*) and herbaceous plants was collected from Joensuu area, Eastern Finland (62°36′N, 29°45′E, 80 m a.s.l.). Roots and soil around the roots were separated from the gross material. Roots were chopped into 1 cm pieces and the rhizosphere soil was sieved with 6-mm sieve. Half of the rhizosphere soil–root mixture was used as inoculum, either as such or autoclaved for the NM plants. The other half was used for preparing a bacterial filtrate, used in addition to the inoculum, as autoclaving kills soil microbes (Parke et al. 1983). Certain soil bacteria are known to directly support the functioning of AM symbiosis, whereas other soil fungi are expected to have little influence in a relatively short-term experiment (Zhang et al. 2024). For the filtrate, the soil–root mix was first incubated overnight in tap water at 5 °C, then sieved through descending sequence of sieves and filter papers, and finally through 4–7 μm paper filter (Schleicher & Schuell, 589/4, Dassel, Germany). The AMF spores are relatively large, 40–320 μm (Souza 2025), while the majority of soil bacteria are less 1.2 μm in diameter (Portillo et al. 2013). Half of the filtrate was applied to the NM treatment and half was autoclaved for use in the AM treatment. The inocula and bacterial filtrates were prepared immediately before the set-up of the experiment and stored at 5 °C for 8–10 days until used. For every pot, 50 mL of soil–root inoculum and a filtrate derived from the same amount of soil–root mixture was applied. Hence, the treatments were:

(i) AM, containing live soil–root inoculum and autoclaved soil filtrate, and,
(ii) NM, containing autoclaved soil–root inoculum and live soil filtrate.

Pots (Ø15 cm, 1.3 L, Schetelig TO 15 D, Vantaa, Finland) were sterilized by keeping them overnight in 70% ethanol. Pieces of filter fabric (sterilized in ethanol) were placed at the bottom to prevent growth media from running out from pots. Pots were filled with peat:perlite mixture up to 2 cm from the top, and then a layer of 50 mL of live or autoclaved inoculum and live or autoclaved filtrate was added over it, and covered with 1 cm layer of peat:perlite mixture.

*Thuja occidentalis* seeds originated from trees grown in Mustila Arboretum, southern Finland (60°43′51″N, 26°25′28″E). Seeds were surface sterilized with H_2_O_2_ (Kilpeläinen et al. 2016) and cold stratified (5 °C) in the dark on water-agar plates (15 g L^−1^ agar with 0.5 g L^−1^ glucose to detect contaminations) for 10 days before plates were moved to germination conditions (22 °C, day length 24 h, Finelectro 18 W 2450 lm). When seed leaves appeared 2 weeks later, the seedlings were transplanted in pots, five seedlings in each pot. The pots were covered by a plastic film to prevent drying of seedlings for the first week after planting.

### Experimental setup

The treatments were two inoculation treatments, mycorrhizal (AM) and NM, seven exposure temperatures (control at 5 °C and six different frost temperatures), and two different recovery conditions (warm 22 °C and cold 10 °C with photoperiod 19 h day/6 h night). The experiment was organized in four blocks. Each block included three parallel pots of each treatment, altogether 84 pots (2 × 7 × 2 × 3), and the group of three parallel pots was considered as experimental unit (replicate). There were five seedlings in each pot (420 seedlings in each block and 1680 seedlings in total). Half of the pots had mycorrhizal seedlings (AM) and another half NM seedlings. Blocks 1, 2 and 3 + 4 were grown at different times, and blocks 3 and 4 were simultaneously grown in separate chamber sections.

All seedlings were first grown for 14 weeks after transplanting in long-day high temperature (LDHT) conditions (daylength 19 h, day/night temperature 22/15 °C, 80% humidity, Conviron GR77, Controlled Environments, Winnipeg, MB, Canada) (Table 1). Incandescent lamps (Airam 60 W, Airam, Kerava, Finland) and fluorescent tubes (Sylvania Cool-white, 215 W, Sylvania, Mississuaga, Canada) were used to provide a photon flux density of ~ 200 μmol m^−2^ s^−1^. Air temperature and relative humidity (Sensirion SHT31-D, Adafruit, USA), photosynthetically active radiation (PAR, SQ-512, Apogee, USA), humidity for each block and soil temperature (DS18B20, Soldata, Denmark) and soil moisture (SoilWatch 10, Pino-tech, Poland) from three randomly selected pots in each block were followed with an external sensor system (Pikkarainen et al. 2022) at 10 min intervals in the growth chambers (Figure S1A available as Supplementary Data at *Tree Physiology* Online). In the first 4 weeks of the growth period, the seedlings were watered with deionized water (Table 1). On week 5, fertilization (NPK 13-7-20 and all other nutrients, GrowHow Puutarhan Kesä, Yara Suomi Oy, Helsinki, Finland) was started with 4 mg N week^−1^ per pot. All other nutrients were in proportion to N. On week 11, fertilization level was increased to 16 mg N week^−1^ per pot, and on week 13, to 32 mg N week^−1^ per pot. During the short-day and freezing conditions fertilization was discontinued and continued again when the recovery period with long-day conditions started with 32 mg N week^−1^ per pot until harvesting.

**Table 1:** Growth conditions of the experiment. ‘Photo’ means photoperiod, day/night (d/n), ‘RH’ relative air humidity and ‘F’ fertilization. ‘SDLT’ refers to short-day low-temperature conditions

	Time, weeks	Photo (d/n), h	Temp (d/n), °C	RH, %	F (N mg week^−1^)
Cold stratification	2	0/24	5/5		
Germination	2	24/0	22/22		
Growth period	4	19/6	22/15	80	0
	6	19/6	22/15	80	4
	2	19/6	22/15	80	16
	2	19/6	22/15	80	32
Frost hardening	1	12/12	16/10	80	0
(SDLT)	2	6/19	6/3	80	0
5 °C or frost exposure −5 °C, −12 °C, −18 °C, −26 °C, −32 °C or –45 °C^1^					
Frost recovery	2	19/6	22/15 or 10/4	80	32

^1^For Block 1 frost treatment temperatures of +5 °C, −2 °C, −5 °C, −12 °C, −18 °C, −30 °C or −40 °C were used.

In the week 15 from planting seedlings to pots, the conditions were changed to short-day low-temperature (SDLT) hardening regime. There was first daylength 12 h and day/night temperature 16/10 °C for 1 week, and then daylength 6 h and day/night temperature 6/3 °C for 2 weeks (Table 1, Figure S1 available as Supplementary Data at *Tree Physiology* Online). After the SDLT conditions, pots were randomly assigned to six different frost exposure conditions or to a control exposure (5 °C). Whole-plant freezing tests in darkness were conducted in three programmable chambers (ARC 300/−55 + 20, Arctest, Espoo, Finland). In the first block, frost temperatures used were 5 °C, −2 °C, −5 °C, −12 °C, −18 °C, −30 °C and −40 °C (Figure S2A available as Supplementary Data at *Tree Physiology* Online), but as the frost tolerance of *T. occidentalis* was greater than expected, exposure temperatures were slightly modified for the remaining blocks to 5 °C, −5 °C, −12 °C, −18 °C, −26 °C, −32 °C and −45 °C (Figure S2B available as Supplementary Data at *Tree Physiology* Online). The data from the temperatures −2 °C, −30 °C and −40 °C were used only for analyzing frost tolerance. Pots were well watered the day before the start of frost exposures. The initial temperature of all exposure temperatures was 5 °C. First, the initial temperature was held for 2 h, then the chamber was cooled at rate 2 °C h^−1^ to −2 °C and maintained 40 h to equalize the temperature in the growth media with the air temperature (data not shown). Then cooling continued at 2 °C h^−1^ until the target temperature, which was maintained for 8 h. The warming rate was 5 °C h^−1^ up to the final temperature + 5 °C. Temperature treatments for each block (1, 2 and 3 + 4) were conducted within a 1-week period so that (i) −12 °C and −26 °C, (ii) 5 °C, −5 °C and −32 °C, (iii) −18 °C and −45 °C, treatments were done in sequence in the same chambers. Length of treatments varied between 42 and 85 h and seedlings were moved to recovery conditions (Table 1) immediately after treatments.

### Relative electrolyte leakage measurements

Immediately after freezing exposure foliage samples were collected for relative electrolyte leakage (REL) analyses (Burr et al. 1990, Radoglou et al. 2007, Korhonen et al. 2019). Six parallel samples within each block were prepared for all mycorrhiza treatment (AM and NM) and frost exposure combinations including the control (5 °C). Each REL sample was prepared using two individual seedlings from each of the three parallel pots. A 1-cm piece was cut from the central part of mature foliage, totalling six pieces from six different seedlings in each REL sample. Then 5 mL of deionized H_2_O was added to the sample tubes, and they were set in a shaker (200 r.p.m.) for 22 h at room temperature. After this, the conductivity of the incubation solution (L_1_) was measured (CDM92-conductivity meter with CDC641T-electrode, Radiometer, Copenhagen, Denmark). The samples were then heat-killed in a water bath (92 °C for 15 min) and shaken again for 22 h before repeating the conductivity measurement (L_2_). The REL was calculated for each sample as:


$$REL=\left(\frac{L_1}{L_2}\right)\ast 100$$

The REL values of the six parallel sample tubes for each exposure temperature were used for fitting a sigmoidal curve (Repo and Lappi 1989) by nonlinear regression (SPSS v. 27, SPPS Inc., Chicago, IL, USA) for both mycorrhiza treatments and all four blocks. The frost hardiness, corresponding to the calculated lethal temperature where 50% of the specimens are killed (LT_50_) was obtained as the inflection point (*C*) of the function:


$$y=\frac{A}{{\left(1+e\right)}^{B\left(C\,-\, x\right)}}+D$$

where y is REL, x is exposure temperature, *A* and *D* define the asymptotes and B is the slope at the inflection point.

Frost recovery treatments and visual damage observation

**Figure 1:**
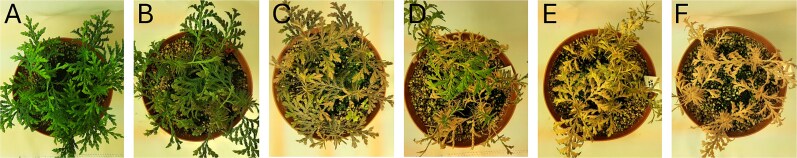
The foliage damage classes 1 to 6 based on color change (A) 0%, (B) 1–25%, (C) 26–50%, (D) 51–75%, (E) 76–99% and (F) 100%.

After the samples for REL were taken, the pots were relocated in the growth chambers in long-day growth conditions to recover from frost exposure, either in warm (22/15 °C) or cold (10/4 °C) temperature conditions (Table 1, Figure S1A and D available as Supplementary Data at *Tree Physiology* Online). After 2 weeks of recovery, visual estimation for damage was conducted for each seedling. Brown foliage was considered as damaged and all shades of green as undamaged. The foliage damage classes 1 to 6 based on proportion of colour change were 0%, 1–25%, 26–50%, 51–75%, 76–99% and 100%, respectively, where 0% stands for no visible damage and 100% stands for entirely brown foliage (Figure 1).

### Above-ground mass and nutrient analyses

At the end of a 2-week recovery period, the above-ground parts of *T. occidentalis* seedlings were cut and dried in paper bags at 45 °C until no further mass loss. Due to the growth habit of young *T. occidentalis* seedlings, biomass contains foliage and young shoot stems formed both pre-freezing and post-freezing. After weighing the dry mass of each seedling, the samples from all seedlings of the three parallel pots within treatments and blocks were pooled. The pooled foliage samples were homogenized with IKA Werke A10 Analytical Grinder (Janke & Kunkel, Staufen, Germany). Nitrogen (N) was analyzed from powdered samples using Vario MAX cube elemental analyzer (Elementar, Langenselbold, Germany). For phosphorus (P), samples were digested in HNO_3_ and H_2_O_2_ in Teflon containers with method based on Epa 3051 in microwave oven (MARS5, CEM, Matthews, NC, USA). Phosphorus levels were determined with iCAP™ 7200 ICP-OES (Thermo Scientific, Waltham, MA, USA). Technical replicates and standard leaf samples were used to check the consistency of the analysis results.

### Mycorrhiza analyses and root mass

After harvesting the above-ground plant parts, the pots from exposure temperatures of 5 °C and −18 °C were sealed in plastic bags and frozen (−21 °C) until further root analyses. All 48 AM pots from exposure temperatures of 5 °C and −18 °C (three pots from each four replicates for both exposure temperatures and both recovery conditions) were analyzed for intensity of AM colonization. To ensure that there was no AM contamination, eight NM pots, one randomly selected pot from each block from exposure temperature of 5 °C (four pots from both recovery temperatures), were investigated for colonization. From half-frozen pots a 1 cm layer from the top was removed and a 2 cm layer below that was separated (Figure S3 available as Supplementary Data at *Tree Physiology* Online). Three sectors, comprising parts of the root systems of each of the five seedlings (Figure S3 available as Supplementary Data at *Tree Physiology* Online), formed a subsample for mycorrhiza analyses. Roots were separated with forceps in tap water from the subsample and cleared and stained according to Grace and Stribley (1991). They were first kept in a 10% potassium hydroxide solution overnight at room temperature, then transferred to an alkaline hydrogen peroxide solution at room temperature, and 20 min later transferred to a 1% hydrochloride dilution for 2 h at room temperature. The staining was done by immersion of the samples for 1 h and a half in a pre-heated solution containing lactic acid, glycerol and methyl blue at 80 °C. Extracellular hyphae, intracellular AM structures (including both arbuscules and Paris-type hyphae), and specialized structures (vesicles and spores) was counted separately in a 1.2 cm gridline drawn in a 90 mm × 15 mm Petri dish under a stereo microscope (Giovannetti and Mosse 1980). AM structures are expressed as % of root length as:


$$\mathrm{AM}\%=\frac{\mathrm{Number}\ \mathrm{of}\ \mathrm{root}\ \mathrm{crossings}\ \mathrm{with}\ \mathrm{AM}\ \mathrm{structures}}{\mathrm{Total}\ \mathrm{number}\ \mathrm{of}\ \mathrm{root}\ \mathrm{crossings}}\times 100$$

Unclear structures were checked with light microscope with different magnifications. Two samples were left out final analyses as they had fewer than 100 root gridline crossings. After microscopic analysis, mycorrhiza subsamples were washed twice in tap water and dried at 45 °C.

Root length in AM subsamples was calculated using grid unit (1.2 cm) and number of total root gridline crossings according to (Tennant 1975):


$$\mathrm{Root}\ \mathrm{length}=\frac{11}{14}\times \mathrm{Total}\ \mathrm{number}\ \mathrm{of}\ \mathrm{root}\ \mathrm{crossings} \times 1.2\ \mathrm{cm}$$

AM colonization per plant was estimated from the sum of all AM structures as % of root length detected from subsamples and estimated root length per plant. The rest of the roots from 5 °C and −18 °C AM as well as corresponding NM pots (total 96 pots) were separated from soil under tap water, dried at 45 °C and weighed. The mass of mycorrhiza subsample roots was added to the total root mass for each pot and divided by number of seedlings within pot to get average root mass per seedling. Total root length for 5 °C and −18 °C AM plants was estimated based on root weight and root length in subsamples and total roots. Specific root length (SRL, m g^−1^) for 5 °C and −18 °C AM was calculated using mass and estimated root length of mycorrhiza subsamples.

### Statistical analyses

All statistical analyses were conducted with SPSS v. 27 (SPPS Inc., Chicago, IL, USA). Full factorial Linear Mixed model with mycorrhiza treatment (M), frost exposure (F) and recovery treatment (R) as fixed factors and block as random factor was used in statistical analyses. If the data did not fulfil the assumptions of the parametric test, transformations were used, and if assumption were still not achieved, corresponding non-parametric tests (Kruskal–Wallis for comparison of multiple groups and Mann–Whitney U for comparison of two groups) were used to test the main effects.

For the lethal temperature for 50% of the samples (LT_50_) there was a single value for each mycorrhiza treatment and block (*n* = 4). The effect of mycorrhizal treatment on LT_50_ value was compared with independent samples t-test. For visual damage observations Mann–Whitney U test was used to compare median values of visual damage recordings between recovery temperatures and mycorrhiza treatments for each exposure temperature separately.

**Figure 2:**
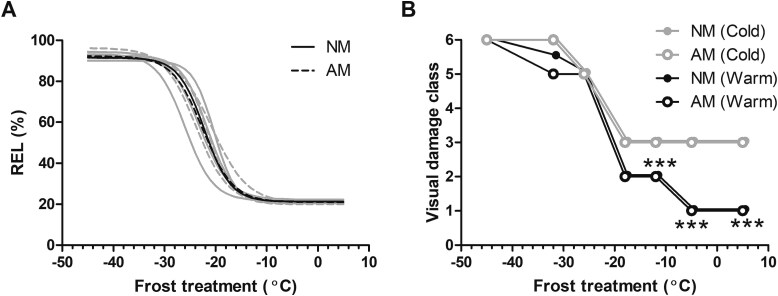
Viability of foliage of NM and AM *T. occidentalis* seedlings after 5 °C or different frost exposures. (A) Black line representing the sigmoidal curve of REL of as function of exposure temperature fitted separately for NM (solid line) and AM (dotted line) treatments (block means, *n* = 4). Grey lines represent individual replicates for NM (solid line) and AM (dotted line). (B) Results for median of visual damage (browning) classified to damage classes from 1 (no damage) to 6 (all foliage damaged). ^***^ refers to statistically significant difference between recovery treatments (‘cold’ and ‘warm’) at *P* < 0.001. Mycorrhiza treatment was not significant.

The shoot mass and nutrient data were analyzed separately for lethal (−26 °C, −32 °C, −45 °C) and non-lethal (5 °C, −5 °C, −12 °C, −18 °C) treatment temperatures, based on the results of REL analysis and visual damage (Figure 2A and B). For seedling mass, and root and fungal characteristics averages of the three pots for each block and treatment combination were calculated. Foliar N and P were analyzed from pooled samples of including all seedlings of the three parallel pots within treatments and blocks and ANOVA was run with frost exposure, recovery treatment and mycorrhiza treatment as fixed factors.

Mass increment during recovery condition was calculated as difference between shoot mass in each non-lethal temperature exposure and the average shoot mass in lethal temperatures (−26 °C, −32 °C, −45 °C).

## Results

### Frost hardiness

**Figure 3:**
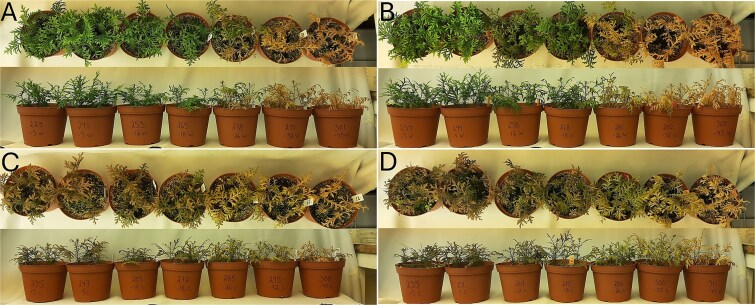
Examples of NM (A, C) and AM (B, D) *T. occidentalis* seedlings treated at 5 °C, −5 °C, −12 °C, −18 °C, −26 °C, −32 °C or −45 °C (from left to right) photographed after 2 weeks recovery period at warm (A, B) or cold (C, D) recovery conditions.

The frost hardiness (LT_50_) of the foliage of *T. occidentalis* did not differ significantly between mycorrhizal treatments (t = −0.245, df = 6, *P* = 0.815) and was −22.9 ± 1.2 °C and −23.4 ± 1.5 °C for NM and AM seedlings, respectively (Figure 2A). Both LT_50_ and the results of visual observations inspected 2 weeks after frost exposures (Figures 2B and 3) indicate that frost damage in the whole plants clearly increased between −18 °C and −26 °C. After the exposures at lower temperatures than −26 °C more than 75% of foliage were recorded as brown (damage classes 5–6).

### Above-ground mass

**Figure 4:**
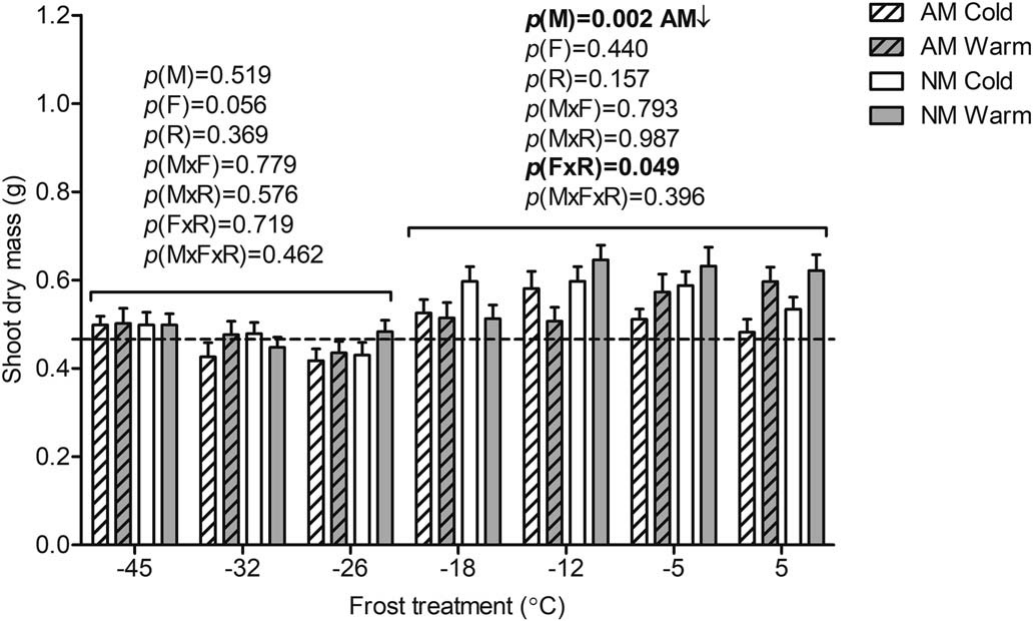
Shoot mass (average, s.e.) of NM and AM *T. occidentalis* (*n* = 3 for −26 °C, −32 °C, −45 °C and *n* = 4 for 5 °C, −5 °C, −12 °C, −18 °C. ‘Cold’ refers to cold recovery (10/4 °C) and ‘warm’ to warm recovery conditions (22/15 °C). The dotted line stands for average shoot mass (0.47 g) for individuals where no growth addition was expected based on visual observation and REL measurements (dead above ground biomass). *P*-values represent results of ANOVA (lethal −26 °C, −32 °C, −45 °C and non-lethal 5 °C, −5 °C, −12 °C, −18 °C exposures tested separately). R stands for recovery treatment, M for mycorrhiza treatment and F for frost exposure. Significant results (*P* < 0.05) are marked with bold, but main effects are marked only when significant interaction is not detected. Arrow (↓) indicates significant decreasing effect of the studied factor on shoot dry mass.

The above-ground mass of *T. occidentalis* seedlings after the lethal frost exposures −26 °C, −32 °C or −45 °C was on average 0.47 ± 0.01 g, and there was no significant effect of frost exposure, recovery temperature or mycorrhizal status and the interactions were not significant (Figure 4). In non-lethal exposures, 5 °C, −5 °C, −12 °C or −18 °C, the main effects of frost exposure or recovery temperature alone were not significant, but there was F × R interaction. The growth during the 2-week recovery after the frost period in seedlings frost treated at −12 °C or −18 °C was not affected by recovery temperature, but after the two mildest temperatures (5 °C, −5 °C) mass increase was on average 15% greater in warm than in cold conditions. Moreover, the main effect of mycorrhiza treatment was significant for the growth recovery after non-lethal frost conditions (Figure 4). In these conditions, average mass increment during recovery period was 0.12 ± 0.02 g for NM seedlings and 0.06 ± 0.02 g for AM seedlings. The interaction between mycorrhiza treatment and frost or recovery treatment was not significant.

### Root mass and root:shoot ratio

**Figure 5:**
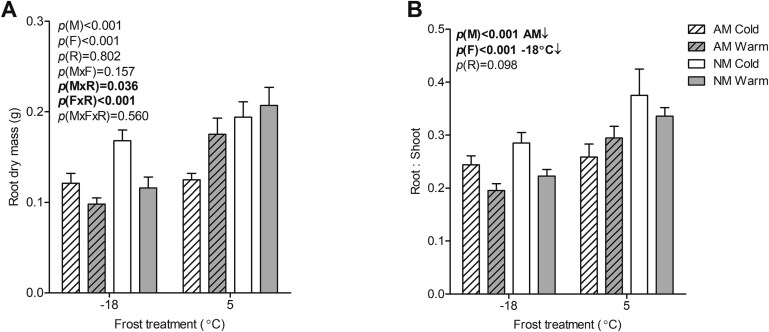
(A) Root mass and (B) root:shoot ratio of NM and AM *T. occidentalis* seedlings after exposures to 5 °C or −18 °C and recovery in warm or cold conditions. R stands for recovery treatment, M for mycorrhiza treatment and F for frost exposure. *P*-values represent significant results of ANOVA for root mass and Kruskal–Wallis test for root:shoot ratio. Significant results (*P* < 0.05) are marked with bold, but main effects are marked only when significant interaction is not detected. Arrows (↓) indicate significant decreasing effect of the studied factor on root:shoot ratio.

For root mass there was significant main effect of frost and mycorrhiza treatment and the interactions M × F, and for R × F were significant. Frost exposure (−18 °C) decreased root mass (Figure 5). In control (5 °C) the recovery temperature had no effect on root mass, but below −18 °C, cold recovery increased root mass (F × R interaction, Figure 5A). In warm recovery temperature NM and AM seedlings had equal root mass, but in cold recovery temperature NM seedlings had 11% higher root mass than AM seedlings (MF × R interaction, Figure 5A).

For root:shoot ratio only the main effects of mycorrhiza and frost treatments were significant and not the interactions. The exposure to −18 °C decreased root:shoot ratio (Figure 5), as for root mass, and root:shoot ratio was greater in NM than in AM seedlings (Figure 5B).

### Mycorrhiza colonization, root length and SRL in AM seedlings

**Figure 6:**
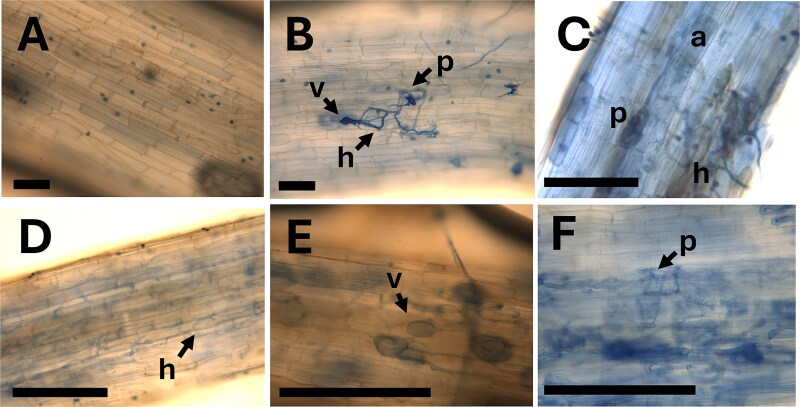
Mycorrhiza structures detected from *T. occidentalis* seedlings. (A) Control plant, no AM structures visible, (B–F) fungal structures coloured with methyl-blue staining (v = vesicle, p = Paris-type hyphae, a = arbuscule, h = hyphae). Reference line corresponds to 20 μm.

**Figure 7:**
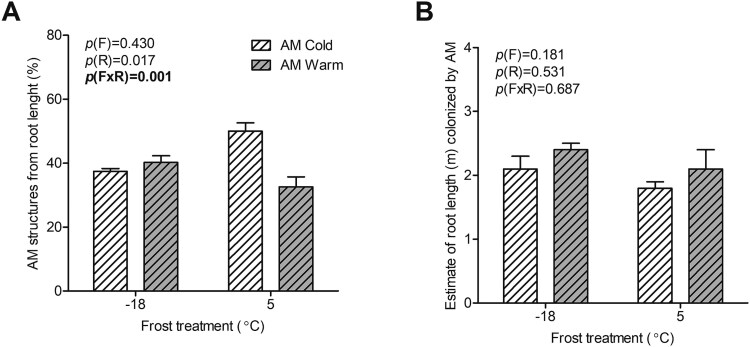
(A) Calculated AM-% of root length and (B) estimate of root length (m) colonized by AM (AM-% × estimated total root length) of *T. occidentalis* seedlings after exposures at 5 °C or −18 °C and recovery in warm or cold conditions. R stands for recovery treatment and F for frost treatment. *P*-values represent significant results of ANOVA (log10-transformed to meet assumptions of parametric test). Significant results (*P* < 0.05) are marked with bold, but main effects are marked only when significant interaction is not detected.

The AM contamination in NM roots was negligible (0.003 ± 0.001% of root length). The most common mycorrhizal structures detected in the AM-inoculated seedlings were extracellular hyphae and Paris-type hyphae (Figure 6, Table 2). Vesicles were seen irregularly, and spores were rare (Table 2). For the AM colonization (% of root length) there was significant main effect of recovery temperature and significant F×R interaction. AM-% was lowest from 5 °C, warm recovery, and highest in pots from 5 °C after cold recovery (F×R interaction, Figure 7A). However, there was also a significant main effect of recovery temperature difference in root length between different treatments. The estimated total root length was on average 25% lower in pots from the cold recovery compared with warm recovery (Table 2). Accordingly, no difference was found in estimate of root length (m) colonized by AM between the treatments (Figure 7B). For SRL there was significant main effect of both frost exposure and recovery temperature. The SRL increased in response to warm recovery treatment, and it was 78% greater after −18 °C than in control treatment (Table 2).

**Table 2:** Specific root length (SRL) and mycorrhiza colonization in AMF inoculated seedlings. *P*-values represent significant results of ANOVA (*P* < 0.05). R stands for recovery treatment (cold or warm) and F for frost treatment (−18 °C or 5 °C). Arrows (↑) indicate significant increasing effect of the studied factor

	−18 °C		5 °C		
	Cold	Warm	Cold	Warm	*P*-values
Estimated total root length (m)^1^	5.0 ± 0.4	5.6 ± 0.5	3.6 ± 0.2	6.0 ± 1.2	*P*(F) = 0.133 *P*(R) = 0.047 W↑ *P*(F×R) = 0.266
SRL (m g^−1^)	45.3 ± 2.1	69.1 ± 2.4	28.8 ± 0.6	35.4 ± 1.9	*P*(F) < 0.001–18 °C↑ *P*(R) = 0.011 W↑ *P*(F×R) = 0.153
AM (% of root length)					
External hyphae^2^	17.2 ± 1.6	17.8 ± 2.3	18.6 ± 1.3	11.7 ± 1.9	*P*(F) = 0.171 *P*(R) = 0.163 *P*(F×R) = 0.077
Intracellular structures^3^	20.1 ± 0.6	23.5 ± 2.8	33.5 ± 4.4	22.2 ± 3.0	*P*(F) = 0.267 *P*(R) = 0.374
Vesicles^3^	1.7 ± 0.6	1.1 ± 0.3	3.7 ± 1.7	0.7 ± 0.2	*P*(F) = 0.085 *P*(R) = 0.050

^1^Log10-transformed, ^2^log10(x + 1)-transformed, ^3^non-parametric.

### Foliar nutrients

**Figure 8:**
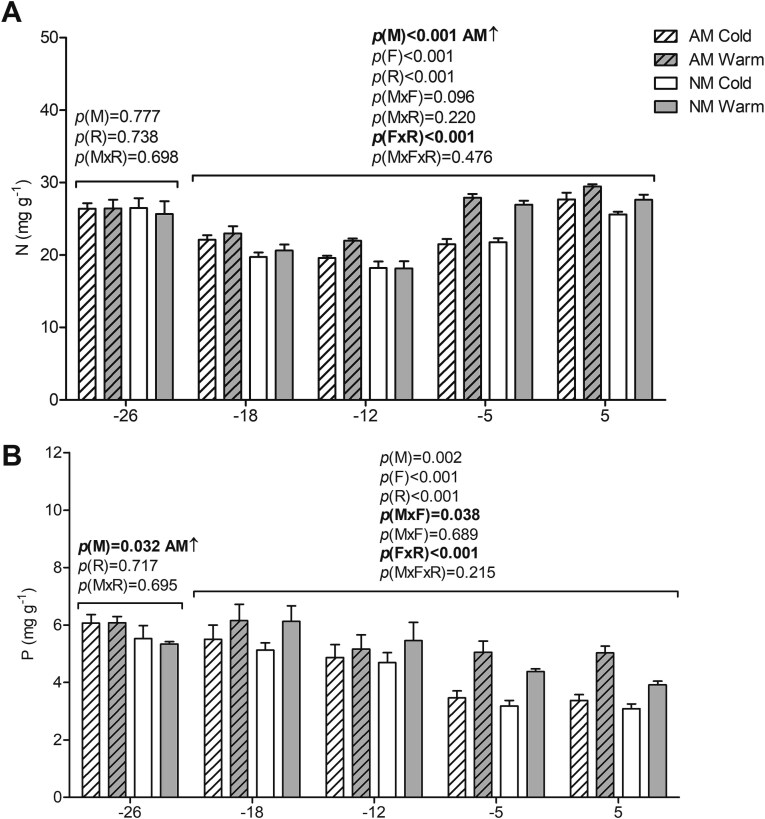
Foliar N (A) and P (B) concentrations of NM and AM *T. occidentalis* (including biomass formed before and after temperature treatment) after exposures from 5 to −26 °C and cold or warm recovery conditions. *P*-values represent significant results of ANOVA (N of −26 °C and P of non-lethal treatments log-transformed to meet assumptions of statistical test). R stands for recovery treatment, M for mycorrhiza treatment and F for frost exposure. Arrows (↑) indicate significant increasing effect of the studied factor on concentrations. Significant results (*P* < 0.05) are marked with bold, but main effects are marked only when significant interaction is not detected.

The nutrient results in the lethal frost exposure −26 °C were used as a proxy of the nutrient status before the frost exposure, because these plants did not grow after the frost exposure, and nutrient leaching would be minimal without rain. In lethal frost exposure (−26 °C)*,* there was significant main effect of mycorrhiza increasing foliar P concentration, while mycorrhiza had no main effect on N concentration (Figure 8).

After non-lethal frost exposures (from −18 °C to 5 °C), the main effect of mycorrhiza, frost and recovery treatments and M×F and F×R interactions on P concentrations were significant. Arbuscular mycorrhiza increased P concentrations after the two mildest exposure temperatures (5 °C and −5 °C) (M×F interaction, Figure 8B, Figures S4B and S5 available as Supplementary Data at *Tree Physiology* Online). In 5 °C and −5 °C P concentration was further increased by warm recovery compared with cold recovery seedlings (F×R interaction, Figure 8B). After non-lethal exposure (from −18 °C to +5 °C) foliar N concentrations were modified by significant main effect for mycorrhiza, frost and recovery treatment and F×R interaction. Nitrogen concentrations were higher in AM plants during the recovery period (Figure 8A, Figure S4A available as Supplementary Data at *Tree Physiology* Online). Warm recovery conditions increased N concentrations compared with cold recovery at least after the two mildest frost conditions (5 °C and −5 °C) (F×R interaction also for N; Figure 8A).

## Discussion

The freezing tolerance (LT_50_) of foliage after whole-plant exposure of young seedlings in the controlled growth chamber experiment was −23 °C. This agrees with Colombo and Raitanen (1991), who concluded that with a hardening pretreatment *T. occidentalis* container seedlings can achieve frost hardiness to −20 °C. Here, the hardening pretreatment before frost exposures was non-freezing, minimum 6/3 °C (day/night). Maximal frost hardiness would be achieved after acclimation to lower temperatures, and in the field, freezing resistance of the foliage up to −45 °C has been shown (Bannister and Neuner 2001). Accordingly, *T. occidentalis* is regarded as a freezing-tolerant species.

Arbuscular mycorrhiza did not affect the needle frost tolerance of *T. occidentalis*, when the samples were taken directly after the frost exposures. Therefore, no rapid physiological adjustments affecting the host plant were detected. Similar results have been obtained earlier when comparing EM and NM *Pinus sylvestris* (Korhonen et al. 2019). Any possible adjustments in the root systems are assessed by the recovery reactions in this study. Direct measurements of root freezing tolerance have methodological problems, because separating roots from soil or growing them without soil can cause similar injuries as freezing (Repo and Ryyppö 2008). This is particularly misleading in comparisons of mycorrhizal and NM roots, as they may be damaged in a different way by pretreatments (Korhonen et al. 2013, 2015). In whole-plant comparisons such as this study, root damage is caused only by the treatments, and therefore they inform about effects on the performance of the plants, including growth and nutrient accumulation. There is a need to develop novel, non-invasive methodologies for information about direct roles of mycorrhizas in the roots. High-pressure flow meters have been used to assess root damage caused by frost (Di et al. 2019, Korhonen et al. 2019), and the method could be developed to suit also small seedlings. Further development of methods based on electrical impedance is also a possibility (Di et al. 2019).

The P concentrations of *T. occidentalis* seedlings before the frost exposures were significantly higher in AM than NM treatment even though peat as growth substrate can, in some cases, suppress AM colonization and host plant growth (Biermann and Linderman 1983). The suppressing effect of peat substrate has been linked for example to lack of P (Biermann and Linderman 1983), naturally low pH and lack of propagules (Perner et al. 2006) in *Sphagnum* peat. However, Anwar et al. (2020) have shown that AM is beneficial for growth and nutrient uptake of *T. occidentalis* on acid peat soil, but only in absence of fertilization. Nutrient accumulation is the clearest sign of a mycorrhizal benefit, and often this leads to increased growth in nutrient limited conditions. Arbuscular mycorrhiza increases especially plant P uptake (Read and Pérez-Moreno 2003), and more recent evidence shows that it has roles also in N uptake (Smith and Smith 2011). Here, fertilization led to N and P concentrations that were not growth-limiting, and the differences detected in concentrations probably did not have significant physiological effects.

In the recovery period, the mycorrhizal plants had higher P concentrations than NM after mild temperature exposures, but not after −12 °C and −18 °C exposures. This agrees with in vitro results by Addy et al. (1998) and the soil study by Lekberg and Koide (2008) who reported metabolically active hyphae for AM after −5 °C. However, after exposure to −12 °C Addy et al. (1998) found reduced number of active hyphae. Damage to hyphae at that temperature could explain why here after −12 °C and −18 °C exposures AM did not affect foliar P concentrations. It should be noted that fungi forming AM are a diverse group and there are species-specific differences in winter survival (Klironomos et al. 2001) and pre-cooling affects survival of AM in freezing temperatures (Addy et al. 1998). Here, the source of mycorrhizal fungi was soil collected from 62°N in late autumn, and fungal species present can thus be expected to be winter tolerant. Moreover, seedlings were autumn treated before frost exposures.

The AM seedlings did not grow more than NM ones during the period before the frost exposures, and after non-lethal frost exposures (from −18 °C to 5 °C) growth was notably reduced as compared with NM seedlings. The young seedlings had a small shoot mass (less than 1 g DW) and relatively high colonization intensity (30–50%) in roots. It has been suggested that these conditions lead to reduced growth because of a high carbon cost of the fungi (Johnson et al. 1997). However, this view is challenged by the fact that plants tend to produce an excess of photosynthates, and environmental stresses often reduce growth before photosynthesis is affected (Prescott et al. 2020). The reasons for the reduced growth of mycorrhizal plants after the frost exposures remain to be explored further, but this study did not show any advantage of the mycorrhizal colonization for the host plant in frost conditions.

We expected that the reduction of growth and nutrient uptake depends on the severity of frost exposure, and that recovery at low temperature would have a negative effect on seedling growth and nutrient levels. Indeed, the recorded root mass and N uptake was larger after milder exposures compared with −18 °C exposure in both AM and NM treatments. Our data suggest that there was non-lethal damage in roots already between −5 °C and −12 °C, which agrees with the result on increasing visual damage after −12 °C exposure in warm conditions. By contrast, the REL results on foliage immediately after the frost exposures did not show an increase until −18 °C, and therefore the delayed response was likely caused by damage in root systems. Accordingly, roots are considered the most vulnerable part of the plant for freezing damage (Hawkins et al. 2021), even though roots are usually not exposed to as low temperatures as foliage. The freezing tolerance of *T. occidentalis* fine roots was earlier reported to be −12 °C (Bigras et al. 2001), and for example fine roots of *Pinus sylvestris* between −6 and −12 °C (Wu et al. 2023). However, root frost hardiness may develop later in the autumn than needle frost hardiness (Di et al. 2019), and therefore the time course of hardening should be taken into account in further studies.

Furthermore, the severity of the frost exposure interacted with recovery temperatures as expected. For N and P uptake, and for root and shoot mass there was a positive effect of warm recovery temperature (22 °C) compared with cold recovery temperature (10 °C) after exposure to −5 °C and 5 °C, but not after the more severe frost exposures. However, AM seedlings exposed to −18 °C had higher SRL than AM seedlings from 5 °C exposure, and warm recovery temperature further increased SRL. This indicates active fine root formation (Ostonen et al. 2007) in −18 °C exposed AM seedlings, replacing frost damaged roots actively with new very fine roots. Similarly, Zhang et al. (2021) showed that AM colonization increased the proportion of fine roots in mechanically damaged root systems. This could mean that even though AM did not increase freezing tolerance of the seedlings, it could have a role in assisting recovery after frost damage. However, the effect was stronger at warm recovery temperature, which agrees with the notion that AM require relatively high temperatures for efficient functioning. Boreal soils remain cool in the spring and early summer conditions, particularly under tree canopies (Tibbett and Cairney 2007). Longer term studies are needed to assess possible roles of mycorrhizas in the recovery in different conditions, and after repeated freezing and thawing.

Temperature during growing season is an important driving force for AM colonization (Cotton et al. 2015). Earlier studies have shown lower AM colonization when plants were grown at 15 °C/14 °C as compared with 23 °C/20 °C (Liu et al. 2004, Kilpeläinen et al. 2020). However, here the mycorrhizal colonization intensity was slightly higher in cold recovery (10 °C) than warm recovery (22 °C) after control exposure (5 °C). The effect of recovery temperature on colonization intensity could have been stronger if the recovery time had been longer, as response time of different AM species to temperature increase is highly variable (Klironomos et al. 2001, Heinemeyer and Fitter 2004). It is also possible that simultaneous injuries in fine roots affected AM function and colonization either directly or indirectly. Nevertheless, when increment in root length is taken into account, there was no significant difference in total root length with AM colonization between recovery temperatures.

## Conclusion

Based on our result *T. occidentalis* is a freeze-tolerant species, but we did not find support to the hypothesis that AM would increase the freezing tolerance of seedlings. Instead, we found evidence that repairing the damage of AM after freezing could have negative effects at the start of the growth period. Shoot growth was lower in AM seedlings than NM ones after all non-lethal freeze-exposures as well as the control (5 °C). Furthermore, AM increased P concentration in foliage only after the mildest temperature exposures (5 °C and −5 °C). Seedlings of a species adapted to AM symbiosis need functional mycorrhizas to be able to compete for nutrients. Our results from the controlled chamber experiment suggest that the function of arbuscular mycorrhiza suffers already in mild winter conditions. Although the results cannot be directly interpreted in terms of field conditions and mature trees, yet they support our hypothesis that temperature is one of the factors limiting distribution of AM-forming plant species in northern area, as suggested by Lehto and Zwiazek (2011) and Kilpeläinen et al. (2016, 2020). In Finland, forest soil temperature under at least 30 cm of snow cover is seldom below −1.5 °C (Sutinen et al. 2008), but it drops significantly lower if snow cover is lacking. An increase in average temperatures in Northern area by global warming is predicted to be highest in winter months putting stable snow cover formation at risk (Mikkonen et al. 2015). Repeated freezing and thawing take place frequently in many boreal soils, particularly in the topsoil where mycorrhizas are mostly located. Therefore, based on our results, *T. occidentalis* roots are likely to be vulnerable to frost damage in future climate.

## Supplementary Material

Supporting_information_Freezing_tolerance_and_recovery_of_Thuja_occidentalis_tpag048

## Data Availability

The data underlying this article will be shared on reasonable request to the corresponding author.
