# Supplementary material for: Freezing tolerance and recovery of arbuscular-mycorrhizal and non-mycorrhizal Thuja occidentalis
Source: Tree Physiol. 2026 Apr 23;46(6):tpag048. doi: 10.1093/treephys/tpag048 (PMC13245403; doi:10.1093/treephys/tpag048)
Supplement: Supporting_information_Freezing_tolerance_and_recovery_of_Thuja_occidentalis_tpag048 [file supporting_information_freezing_tolerance_and_recovery_of_thuja_occidentalis_tpag048.docx]

Supplementary material

**Freezing tolerance and recovery of arbuscular-mycorrhizal and non-mycorrhizal Thuja occidentalis**

Virjamo Virpi ^1^, Repo Tapani ^2^, Lehto Tarja ^1,2,3^

^1^ School of Forest Sciences, University of Eastern Finland, Joensuu, Finland

^2^ Natural Resources Institute Finland, Joensuu, Finland

^3^Present address: Natural Resources Institute Finland, Helsinki, Finland


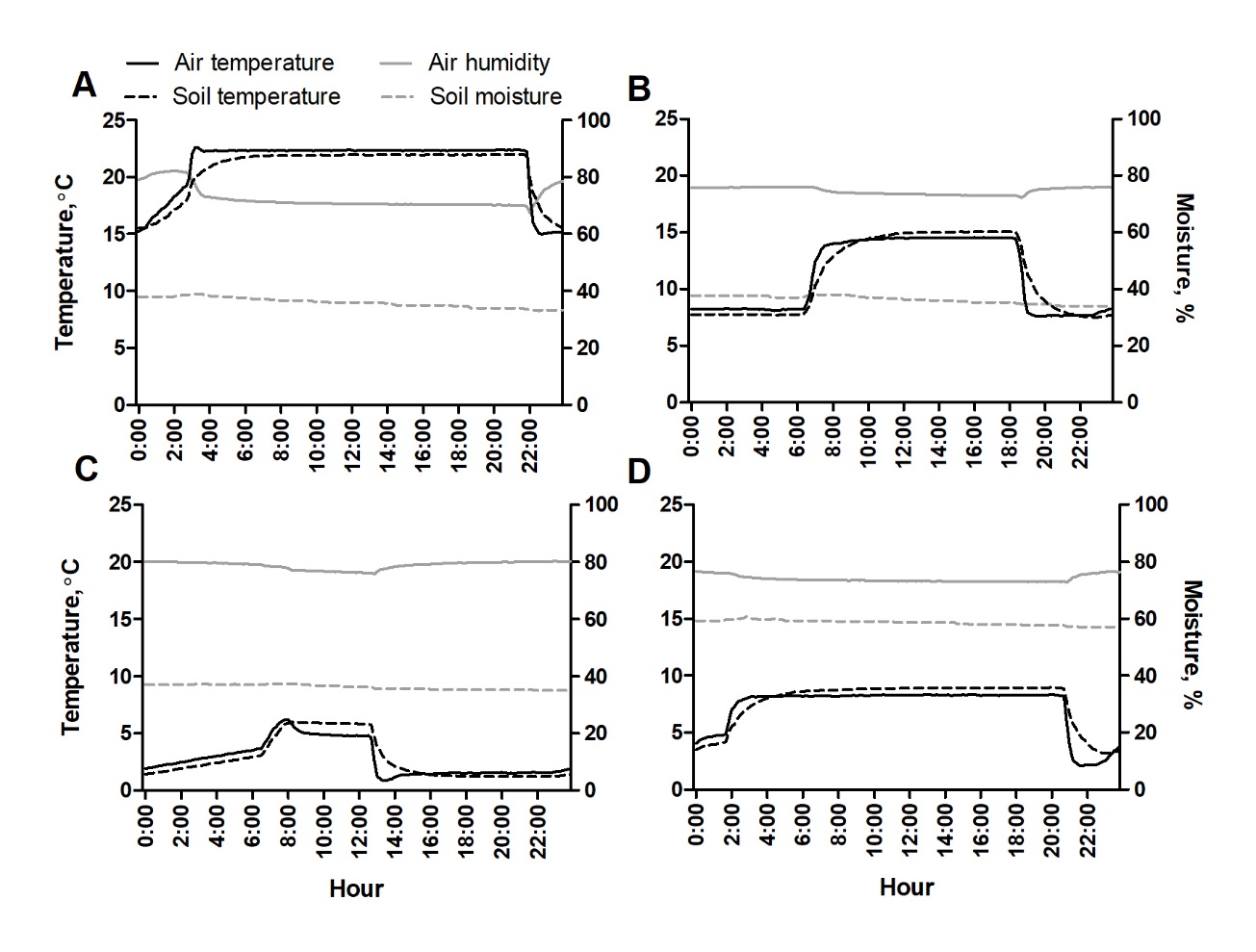


**Supplementary figure 1.** Examples of daily temperature changes and air humidity and soil moisture during A) long day high temperature conditions (LDHT) during growing period, B) and C) two consecutive short day low temperature conditions (SDLT) during frost hardening treatment, and A) and D) conditions for frost recovery periods (‘Warm’ in A and ‘Cold’ in D).


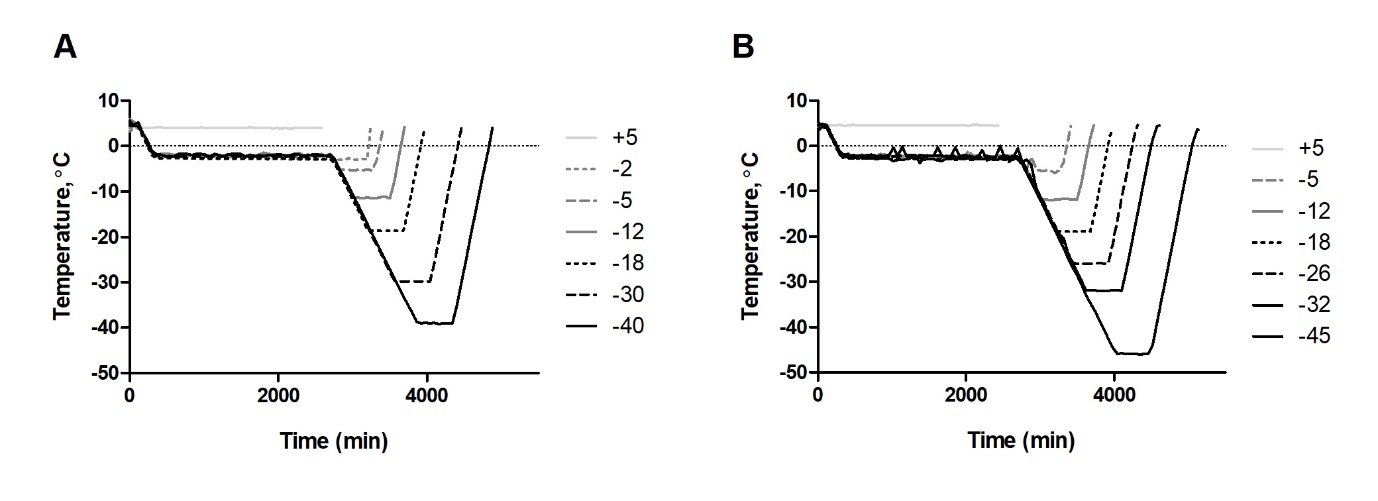


**Supplementary figure 2.** Schematic presentations of measured freezing exposure temperatures for A) block 1 and B) block 2,3 and 4.


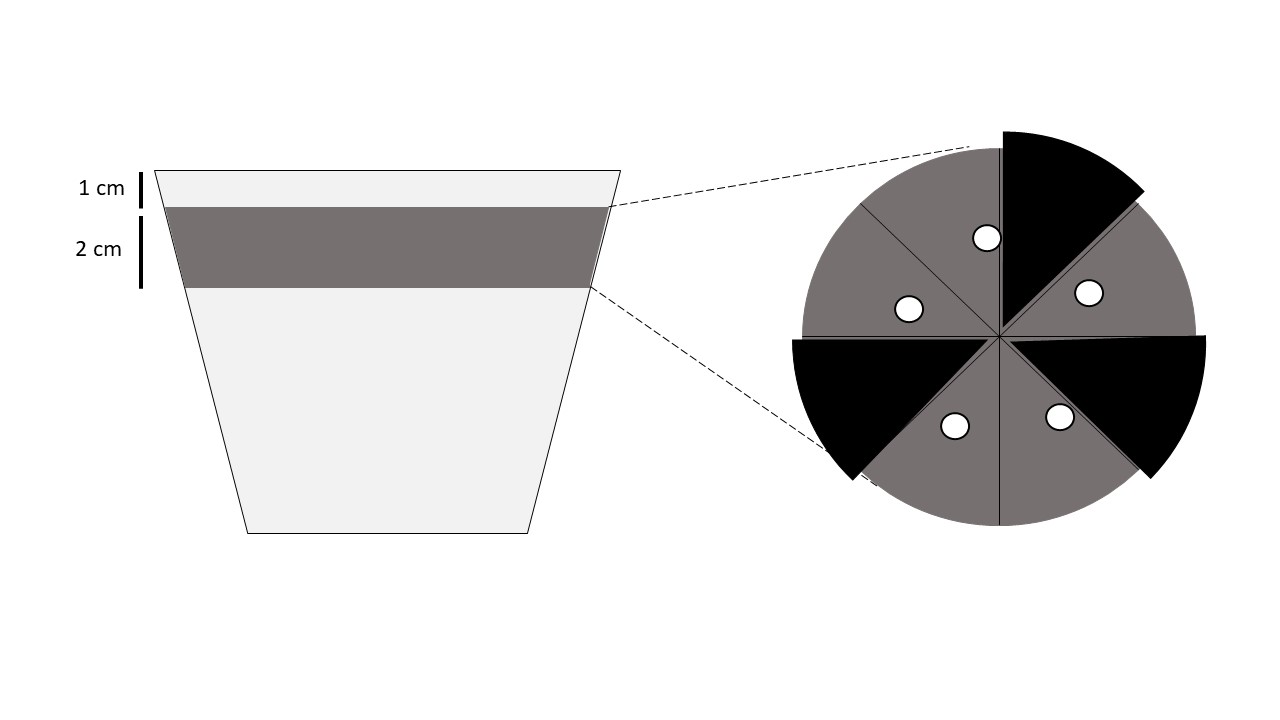


**Supplementary figure 3.** Schematic presentation of subsamples (marked as black sectors) used in mycorrhiza and root length analysis.


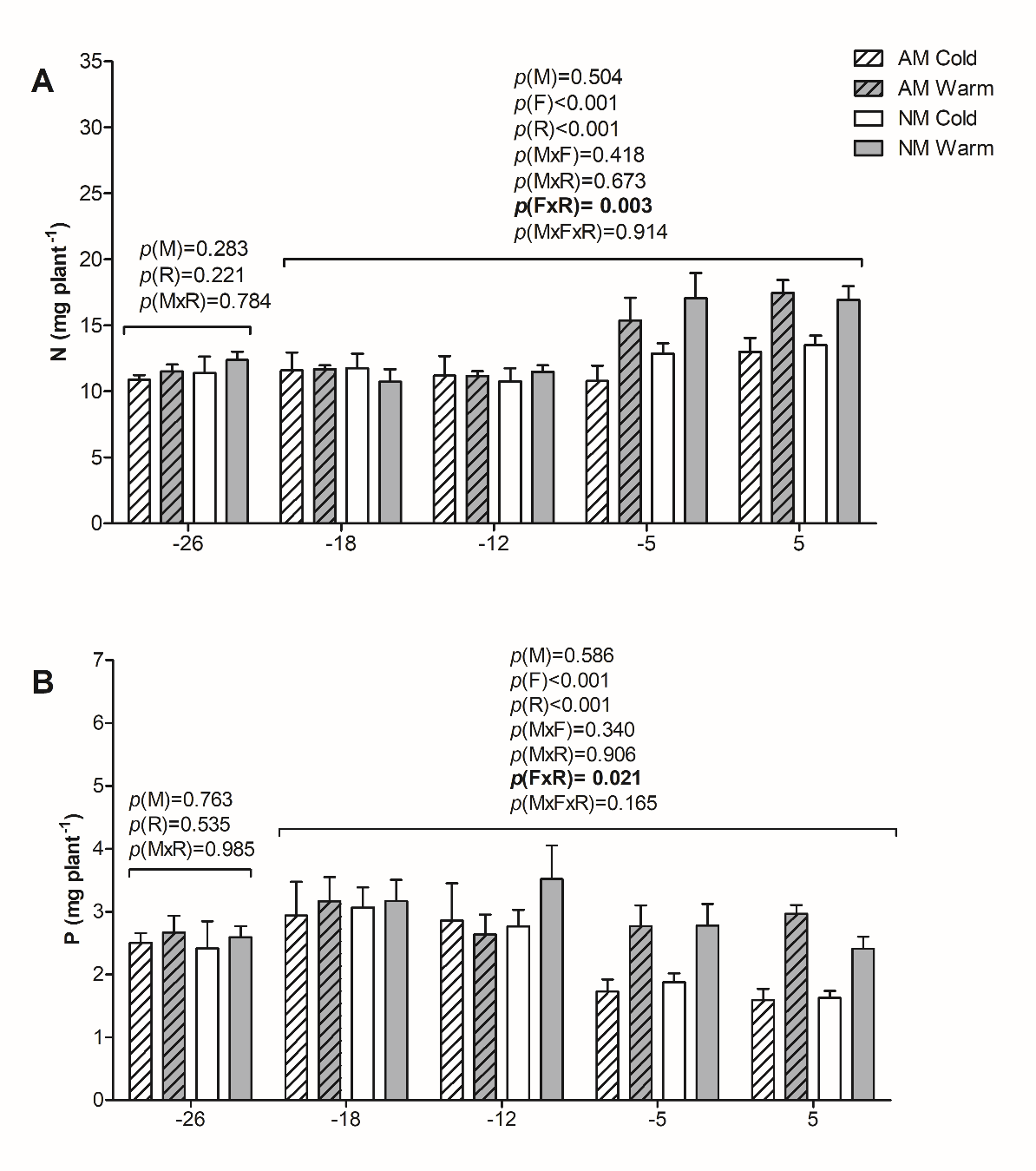


**Supplementary figure 4.** A) Foliar N, and B) P content of non-mycorrhizal (NM) and arbuscular mycorrhizal (AM) *T. occidentalis* after exposures from 5 to -26 °C and cold or warm recovery conditions. *p*-values represent significant results of ANOVA. R stands for recovery treatment, M for mycorrhiza treatment and F for frost exposure. Growth form of young *T. occidentalis* seedlings does not have clearly separated stem and foliage. For this reason, nutrient contents calculated based on foliage biomass and nutrient concentrations might have inaccuracies.

**
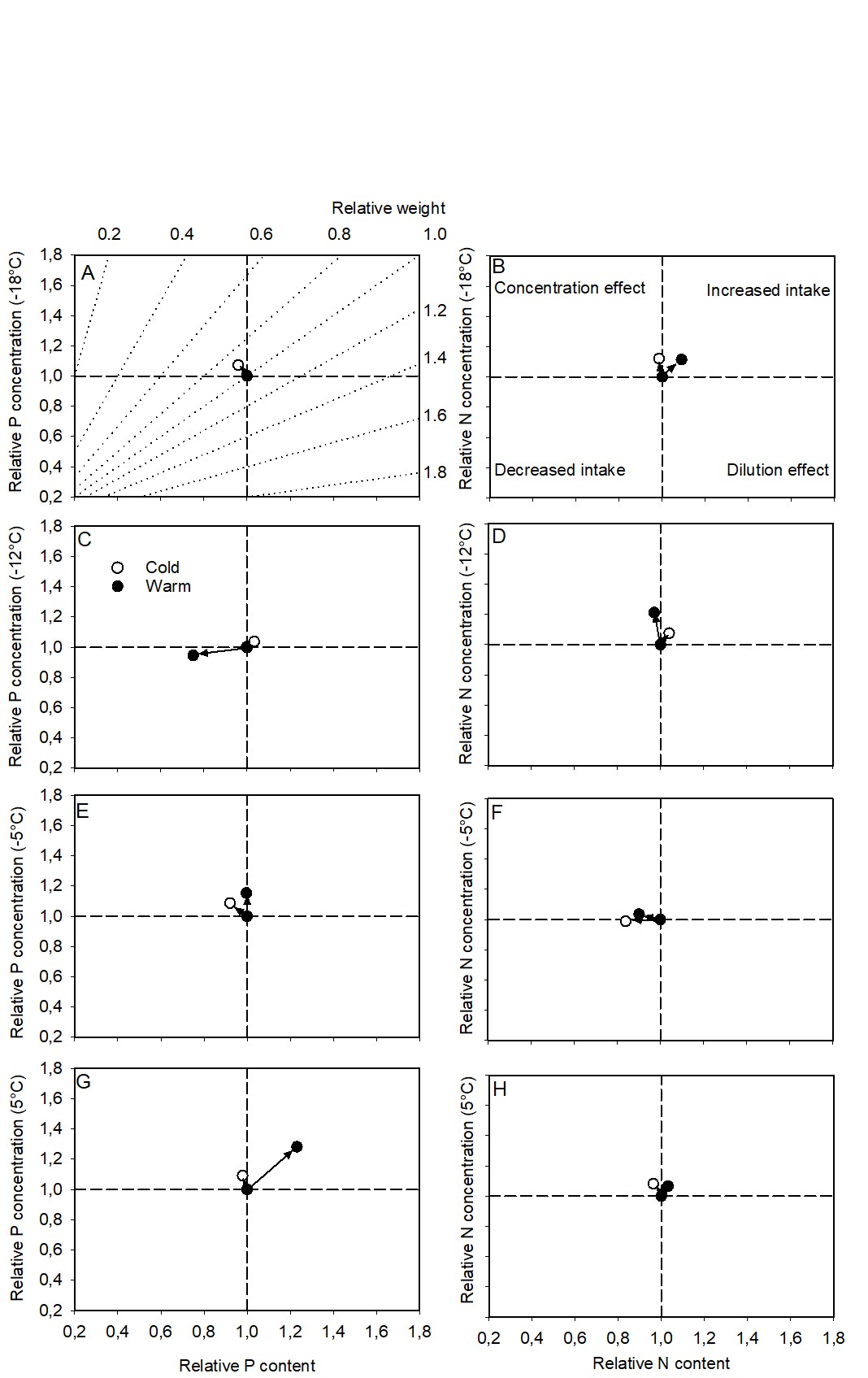
**

**Supplementary figure 5.** Graphical vector analysis (GVA) was used to clarify the effect of variation in seedling size to variation in nutrient concentrations. Effect of AM after exposure in 5°C, -5°C, -12°C, -18°C presented with graphical vector analyses for P (A, C, E, G) and N (B, D, F, H). Results for warm recovery with closed symbols and for the cold recovery with open symbols. The direction of the arrow shows change in relative content: relative concentration ratio compared to reference point (NM). Direction of change in GVA is interpreted as result of increased uptake, decreased uptake, concentration effect or dilution effect (B) and can be used to separate effect on nutrient concentration caused by differences in growth or in function.The NM treatment (separately for each non-lethal growth condition 5°C, -5°C, -12°C, -18°C) was taken as control point (x, y, and z = 1,1,1) to separate effect of AM on nutrient uptake. Relative values for content (x), concentration (y), and biomass (z) were calculated according Haage and Rose (1995).

*Haase DL, Rose R (1995) Vector analysis and its use for interpreting plant nutrient shifts in response of silvicultural treatments. For Sci 41:54–66*

**Supplementary table 1.** Statistical results corresponding *p*-values presented in fig 4, 5,7, 8 and table 2 for non-mycorrhizal (NM) and arbuscular mycorrhizal (AM) *T. occidentalis* after exposures from 5 to -45 °C and cold or warm recovery conditions. R stands for recovery treatment, M for mycorrhiza treatment and F for frost exposure. Lethal conditions refer treatments below -18°C, and non-lethal treatments to 5 to -18 °C.

|  | **Denominator** | **F-value** | | | | | | |
| --- | --- | --- | --- | --- | --- | --- | --- | --- |
|  | **df** | **M** | **F** | **R** | **MxF** | **MxR** | **FxR** | **MxFxR** |
| **N (lethal)** | 6.000 | 0.088 |  | 0.122 |  | 0.165 |  |  |
| **N (non-lethal)** | 45.000 | 28.539 | 110.771 | 51.231 | 2.244 | 1.548 | 11.352 | 0.847 |
| **P (lethal)** | 6.000 | 7.726 |  | 0.145 |  | 0.170 |  |  |
| **P (non-lethal)^1^** | 45.004 | 10.815 | 73.507 | 101.760 | 3.050 | 0.162 | 7.867 | 1.551 |
| **Shoot biomass (non-lethal)** | 173.000 | 9.428 | 0.905 | 2.020 | 0.344 | 0.000 | 2.671 | 0.997 |
| **Shoot biomass (lethal)** | 94.000 | 0.418 | 2.979 | 0.813 | 0.250 | 0.350 | 0.331 | 0.778 |
| **Estimated total root length (m)^1^** | 12.000 |  | 2.597 | 4.922 |  |  | 1.363 |  |
| **SRL** | 41.000 |  | 22.872 | 7.193 |  |  | 2.120 |  |
| **Hyphae^2^** | 42.000 |  | 1.940 | 2.015 |  |  | 3.297 |  |
| **AM % ^1^** | 38,853 |  | 0.635 | 6.263 |  |  | 13.528 |  |
| **Estimate of root lenght colonized by AM** | 41.000 |  | 1.851 | 0.399 |  |  | 0.164 |  |
| **Root biomass** | 85.000 | 24.140 | 29.339 | 0.063 | 2.035 | 4.527 | 20.494 | 0.343 |
| **P content (lethal)** | 6.000 | 0.100 |  | 0.433 |  | 0.000 |  |  |
| **P content (non-lethal)** | 45.004 | 0.302 | 13.983 | 24.810 | 1.148 | 0.014 | 3.566 | 1.778 |
| **N content (lethal)** | 6.000 | 1.388 |  | 1.861 |  | 0.082 |  |  |
| **N plant (non-lethal)** | 45.000 | 0.453 | 14.357 | 15.374 | 0.963 | 0.180 | 5.548 | 0.173 |
|  |  | **U-value** | **df, H-value** | **U-value** |  |  |  |  |
| **Root:shoot^3^** |  | 668.000 | 1; 20.976 | 926.000 |  |  |  |  |
| **Intracellular structures^3^** |  |  | 1; 3.830 | 214.000 |  |  |  |  |
| **Vesicles^3^** |  |  | 1; 0.791 | 187.000 |  |  |  |  |

**^1^** log10-transformed, **^2^** log10(x+1)-transformed, **^3^** non-parametric
